# Efficacy of Non‐Ablative Fractional Laser Combined With Tranexamic Acid in Melasma Treatment: A Randomized Split‐Face Trial

**DOI:** 10.1111/jocd.70384

**Published:** 2025-08-08

**Authors:** Amirhossein Rahimnia, Motahareh Hosnian, Amir houshang Ehsani, Pedram Nourmohammadpour, Zahra Shadlou, Mina Koohian Mohammadabadi, Ala Ehsani

**Affiliations:** ^1^ Department of Dermatology, Razi Hospital Tehran University of Medical Sciences Tehran Iran; ^2^ Autoimmune Bullous Diseases Research Center, Razi Hospital Tehran University of Medical Sciences Tehran Iran; ^3^ School of Medicine Tehran University of Medical Sciences Tehran Iran; ^4^ School of Medicine Iran University of Medical Sciences Tehran Iran; ^5^ Skin Research Center Shahid Beheshti University of Medical Sciences Tehran Iran; ^6^ Student Research Committee, Faculty of Medicine Shahid Beheshti University of Medical Sciences Tehran Iran

**Keywords:** erbium: YAG laser, fractional photothermolysis, melasma, non‐ablative lasers, topical therapy

## Abstract

**Introduction:**

The non‐ablative fractionated erbium glass laser (NAFL) 1540 nm combined with topical tranexamic acid (TXA) is a novel therapeutic approach for melasma. However, the efficacy and safety of this combination remain controversial. This study aims to evaluate the effectiveness and safety of NAFL combined with TXA in treating melasma.

**Methods:**

A randomized controlled trial with a split‐face design was conducted on 27 participants with symmetric melasma. All participants applied 5% TXA topically twice daily to their entire face. One side of the face underwent three NAFL sessions combined with TXA at 4‐week intervals, while the opposite side, treated with TXA alone, served as the control. Outcomes were assessed using the modified melasma area and severity index (m‐MASI), physician global assessment (PGA), and participant satisfaction at the end of treatment, and at one and 3 months after the final laser session.

**Results:**

Both facial sides showed significant m‐MASI score improvement at the end of treatment (*p* < 0.001); however, a significant recurrence was noted in both groups during follow‐up. The reduction in m‐MASI scores was significantly greater for the NAFL plus TXA group than for TXA alone at the end of treatment (*p* = 0.029). However, no significant difference was observed between the groups at the 1‐ and 3‐month follow‐ups (*p* = 0.33 and 0.31, respectively). NAFL plus TXA led to significantly better PGA scores at Week 12 (*p* < 0.001), but this improvement was not sustained during follow‐up (*p* = 0.06 and 0.07, respectively). Participant satisfaction was consistently higher in the NAFL plus TXA group (*p* = 0.001).

**Conclusions:**

The combination of NAFL and topical TXA provides significant short‐term clinical benefits in treating melasma, likely by enhancing TXA penetration into target tissues and improving pigmentary clearance. However, its efficacy diminishes over time due to the inevitable recurrence of melasma, necessitating further investigation into long‐term maintenance strategies.

## 1 Introduction

Melasma is one of the most common and refractory hyperpigmentation skin disorders, typically presenting as irregular light to dark brown macules or patches on the face, and more frequently affecting individuals with darker skin types [1]. Although this skin condition is more prevalent among women—particularly during pregnancy—men can also be affected [2]. Notably, the overall prevalence of melasma varies widely, ranging from 1% to 50%, depending on the ethnicity and geographic location of the population [3].

Although the exact etiology of melasma remains unknown, it is believed to result from a complex interplay of genetic, hormonal, and environmental factors, including sun exposure, family history, pregnancy, thyroid dysfunction, and certain medications [4, 5]. These multifactorial influences contribute to the chronic and often relapsing nature of the condition, complicating effective treatment.

Current treatment strategies primarily focus on sun‐protective measures and the application of topical agents, such as hydroquinone, non‐hydroquinone‐based creams, tretinoin, and corticosteroids [6]. Among the emerging therapies, tranexamic acid (TXA)—a synthetic derivative of the amino acid lysine and a plasmin inhibitor—has shown considerable promise. TXA acts by inhibiting UV‐induced plasmin activity, suppressing melanin synthesis through the downregulation of paracrine melanogenic factors, and reducing angiogenesis by inhibiting vascular endothelial growth factor (VEGF) and endothelin‐1‐induced vascular changes, thereby decreasing visible pigmentation [7].

TXA is available in topical, intralesional, and oral formulations [8]. However, it is important to note that topical treatments may be insufficient in some participants due to limited therapeutic response, slow clinical improvement, or adverse effects such as erythema, skin irritation, and post‐inflammatory hyperpigmentation [9].

In contrast, laser and light therapies represent tertiary treatment options, particularly beneficial for participants who do not respond to topical agents or who seek more rapid results. Recently, non‐ablative fractionated erbium glass lasers (NAFLs) have gained attention as a promising modality for melasma treatment. These devices target hydrous tissue and create columns of thermal coagulative damage within the dermis, while remaining below the threshold for ablation [10]. Importantly, the stratum corneum remains intact during treatment, and no visible wound is formed. Among these, fractional 1550/1540 nm non‐ablative laser therapy is the only FDA‐approved laser‐based intervention for melasma [11].

Specifically, NAFL at 1540 nm utilizes mid‐infrared wavelengths that bypass the epidermis and penetrate the dermal‐epidermal junction to the mid‐reticular dermis—reaching depths of up to approximately 1500 μm—to stimulate neocollagenesis and dermal remodeling. However, short‐term adverse effects, including pain, erythema, and swelling, are commonly observed following this procedure [12].

Given these considerations, the rationale for our study arises from the need to address ongoing limitations in melasma management. While TXA offers a convenient and non‐invasive approach, its efficacy may be restricted by limited dermal absorption. Conversely, NAFL at 1540 nm provides targeted photothermal effects that can improve dermal remodeling and reduce pigmentation, yet there is a lack of robust data on its combined use with topical agents like TXA. Therefore, this study aims to assess the combined effect of NAFL and TXA in comparison to TXA alone, with the goal of determining whether their synergistic action can enhance treatment outcomes while maintaining an acceptable safety profile. This approach may ultimately offer a more effective therapeutic strategy for participants with refractory melasma.

## 2 Materials and Methods

### 2.1 Participant Population and Study Design

This single‐blind, randomized controlled trial was conducted on participants with melasma who were referred to Razi Dermatology Hospital, a tertiary dermatologic center, between March 2022 and February 2024. The inclusion criteria comprised adults aged between 18 and 65 years, the presence of symmetric melasma lesions on both sides of the face, and Fitzpatrick skin types I to IV. Participants with any history of vitiligo, pigmentation disorders, keloidal tendencies, coronary heart disease, blood clotting disorders, or active intravascular coagulation were excluded. In addition, pregnancy, breastfeeding, or plans for pregnancy during the study period were considered exclusion criteria.

Furthermore, participants who had used hydroquinone medications, oral or topical retinoids, topical steroids, alpha hydroxy acids, chemical peels, microdermabrasion, dermabrasion, or laser therapy within the past 3 months were excluded. Similarly, those with a history of solarium use or significant sun exposure during the same period were not eligible for inclusion. All participants were required to comply with the study protocol and attend scheduled follow‐up sessions.

### 2.2 Ethical Considerations

This study was conducted in accordance with the principles outlined in the Declaration of Helsinki. Written informed consent was obtained from all participants prior to enrollment. Notably, participants were provided with comprehensive information regarding the study procedures, potential risks, and benefits, and were informed of their right to withdraw at any time. Ethical approval for the study was granted by the Research Ethics Committee of Tehran University of Medical Sciences (approval ID: IR.TUMS.REC.1394.1905), and the trial was registered in the Iranian Registry of Clinical Trials (IRCT) under the registration number IRCT2016011626047N1.

### 2.3 Treatment Protocol

All participants received a topical 5% TXA solution, formulated in a mixture of cold cream and distilled water, which was applied twice daily on both sides of the face. The formulation process was carried out by our hospital's compounding pharmacy team, including licensed pharmacists, who oversaw the accurate preparation and quality control of the cream. The selected concentration of 5% TXA was based on the study by Kanechorn Na Ayuthaya et al. [13], which demonstrated its efficacy and safety in topical use. To prepare the cream, 5 g of pure pharmaceutical‐grade TXA powder were weighed and dissolved in a small amount of distilled water to form an aqueous solution. This solution was gradually mixed into approximately 90–95 g of a commercially available, fragrance‐free cold cream base with continuous gentle stirring until a uniform and smooth consistency was achieved. Additional distilled water was added as needed to reach the final weight of 100 g and ensure complete homogenization. The resulting cream was stirred thoroughly until the desired texture was obtained and then stored in suitable opaque containers to preserve stability and prevent degradation. The final product contained a TXA concentration of 5% weight/weight (w/w).

For the intervention group, one‐half of each participant's face was randomly assigned to undergo treatment with a non‐ablative fractional erbium glass 1540 nm laser (Palomar Lux 1540 Laser), administered in three sessions at 4‐week intervals.

Topical anesthesia with lidocaine 2.5% combined with prilocaine 2.5% ointment was applied prior to laser treatment. The procedure was carried out using a 15 mm handpiece, a pulse duration of 15 milliseconds, two to three passes, and an energy level ranging from 15 to 20 mJ/MB. Fluence levels were set between 15 and 20 J/cm^2^ and adjusted based on treatment response to achieve mild erythema.

Following laser treatment, participants were instructed to apply zinc oxide ointment for 5 days. Moreover, they were advised to use a sunscreen (SPF ≥ 50) and a moisturizer containing Eucerin without any brightening agents for the 12‐week duration of the study.

### 2.4 Efficacy Assessment

The efficacy of the treatments was evaluated using standardized photographs captured at each visit with the VisioFace Quick (Courage and Khazaka, Germany) imaging system, ensuring consistency in zoom settings, distance, and lighting conditions. Photographs were obtained from frontal, right lateral, and left lateral perspectives.

To quantify melasma severity, the modified melasma area and severity index (m‐MASI) score was used at baseline, at Week 12 (end of treatment), and at the first‐ and third‐months post‐treatment. This scoring system accounts for both the area (A) and the darkness (D) of melasma lesions using the following formula:

The right forehead (RF), right malar region (RM), and right chin (RC) correspond to 15%, 30%, and 5% of the total facial surface area, respectively. These same regions on the left side complete the full facial assessment. Area involvement was scored as follows: 0 (no involvement), 1 (< 10%), 2 (10%–29%), 3 (30%–49%), 4 (50%–69%), 5 (70%–89%), and 6 (90%–100%). The m‐MASI score for each side was calculated using the formula:

$$\begin{array}{cc} m-\text{MASI score}\;=\; & 0.15\times A\;\left(\text{forehead}\right)\times D\;\left(\text{forehead}\right)\\ & +0.3\times A\;\left(\text{malar}\right)\times D\;\left(\text{malar}\right)+0.05\\ & \times A\;\left(\text{chin}\right)\times D\;\left(\text{left chin}\right) \end{array}$$

In addition, the Physician's Global Assessment (PGA) was performed by a blinded dermatologist and categorized according to the percentage reduction in pigmentation: excellent (> 75%), marked (50%–75%), good (25%–49%), fair (< 25%), or worsened (0%). Participant satisfaction was documented at the end of the study. The final assessment of clinical improvement was conducted at baseline, Week 12, and the first‐ and third‐months post‐treatment.

### 2.5 Statistical Analysis

All statistical analyses were performed using SPSS version 28.0 (IBM Corp., Armonk, NY, USA). Continuous variables were analyzed using repeated measures ANOVA and Student's *t*‐test, whereas categorical variables were compared using the chi‐square (*χ* ^2^) test. A *p*‐value of < 0.05 was considered statistically significant.

## 3 Results

A total of 36 cases of melasma were assessed for eligibility. During the study, 7 participants were unable to complete the treatment due to noncompliance, and 2 participants experienced unexpected pregnancies. Consequently, 9 participants (25%) were excluded from the study, and the data of 27 participants (75%) were included in the final analysis. The study design entailed evaluations at four specific time points: baseline, at the end of the 3‐month treatment period, 1 month post‐treatment, and 3 months post‐treatment.

Among the 27 analyzed participants, 5 were men (17.5%) and 22 were women (81.5%), with a mean age of 38.5 years (range: 23–65 years, SD: 12.06). Notably, the majority (66.66%) had Fitzpatrick skin type III, and the remainder had type IV. In addition, 68.2% of the female participants reported a history of pregnancy. To facilitate intra‐individual comparison, a split‐face design was employed: one side of the face received both topical 5% TXA and non‐ablative fractionated erbium glass 1540 nm laser (Group A), while the contralateral side received only topical TXA (Group B). Thus, 27 facial sides were analyzed in each group. Table 1 presents the clinicodemographic data.

**TABLE 1:** Participant clinicodemographic data.

Subject demographics		Values
Age (year)		38.5 ± 12.06
Sex	Male	22 (81.5%)
	Female	5 (18.5%)
Fitzpatrick skin types	I	0 (0.0%)
	II	0 (0.0%)
	III	18 (66.66%)
	IV	9 (33.33%)
Frequency of pregnancy	0	7 (31.75%)
	1	4 (18.2%)
	2	9 (40.9%)
	3	2 (9.1%)

*Note:* Data are shown in number (%) or mean ± SD.

### 3.1 Modified Melasma Area and Severity Index

Initially, the efficacy of the treatment was evaluated separately in each group. The NAFL plus TXA‐treated side (Group A) had a baseline mean m‐MASI score of 4.38 ± 2.86, which significantly decreased to 2.16 ± 1.9 at Week 12 (*p* < 0.001). This reduction demonstrates the efficacy of the combined treatment in improving melasma during the treatment period. However, following the discontinuation of treatment, the m‐MASI score showed a significant upward trend, increasing to 3.1 ± 2.94 1 month post‐treatment (*p* = 0.014) and to 3.42 ± 3.02 3 months post‐treatment (*p* = 0.002). These results indicate a clear recurrence of melasma lesions after stopping therapy. Overall, the combination of NAFL and topical TXA was effective in reducing m‐MASI scores, but its effects diminished over time due to recurrence.

The TXA‐only treated side (Group B) exhibited similar findings. The mean m‐MASI score in this group decreased from a baseline of 4.03 ± 2.9 to 2.75 ± 2.63 at Week 12 (*p* < 0.001), reflecting a significant improvement during the treatment period. However, after treatment cessation, a significant increase in the m‐MASI score was observed, reaching 3.29 ± 2.9 1 month post‐treatment (*p* = 0.001) and 3.6 ± 2.85 at 3 months post‐treatment (*p* < 0.001). These results suggest that while topical TXA alone is effective during active treatment, melasma recurrence occurs after stopping therapy, similar to Group A. In both groups, the recurrence of melasma was significant compared to the end of the treatment period, but there was no notable difference between the two. In other words, the laser had no effect on reducing the recurrence of melasma after the cessation of topical TXA.

#### 3.1.1 Comparison Between Group A and Group B

**FIGURE 1:**
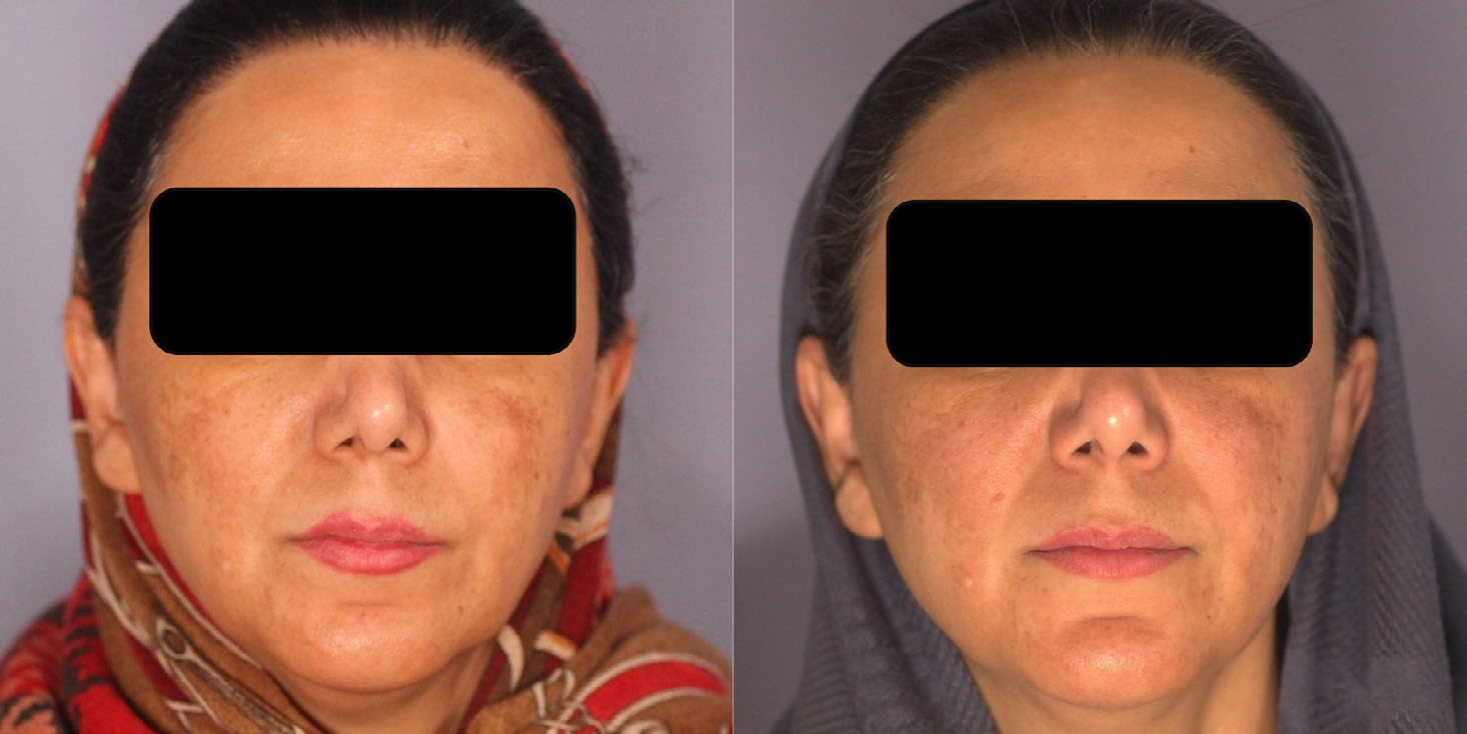
Before (left) and after (right) treatment, showing better response on the right side (left in the image—NAFL + TXA).

**FIGURE 2:**
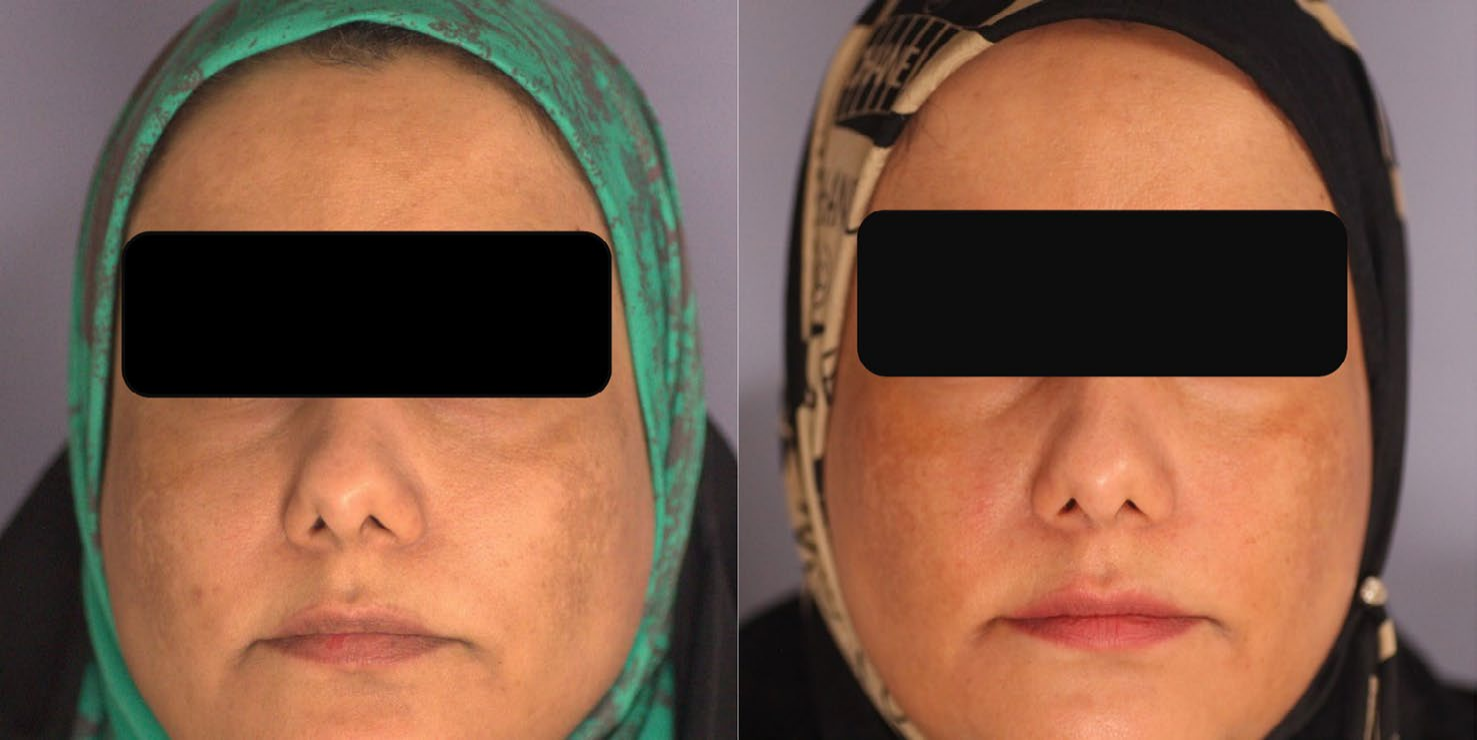
Before (left) and after (right) treatment, showing better response on the left side (right in the image—NAFL + TXA).

At the end of the 12‐week treatment period, there was a significant difference in m‐MASI scores between the two treatment groups, with Group A (2.16 ± 1.9) showing greater improvement compared to Group B (2.75 ± 2.63) (*p* = 0.029). However, this difference was not statistically significant at 1 month (*p* = 0.33) or 3 months (*p* = 0.31) post‐treatment, indicating that both groups experienced comparable levels of recurrence over time (Figures 1 and 2).

The detailed comparison of m‐MASI scores between the two groups at baseline, Week 12, 1 month post‐treatment, and 3 months post‐treatment is presented in Table 2.

**TABLE 2:** The comparison of m‐MASI scores in NAFL plus TXA‐treated side and TXA alone‐treated side (*n* = 27).

	NAFL plus TXA‐treated side	TXA alone‐ treated side	*p*
Baseline	4.38 ± 2.86	4.03 ± 2.9	0.01
Week 12	2.16 ± 1.9	2.75 ± 2.63	0.029
1‐Month post‐treatment	3.1 ± 2.94	3.29 ± 2.9	0.33
3‐Month post‐treatment	3.42 ± 3.02	3.6 ± 2.85	0.31

**FIGURE 3:**
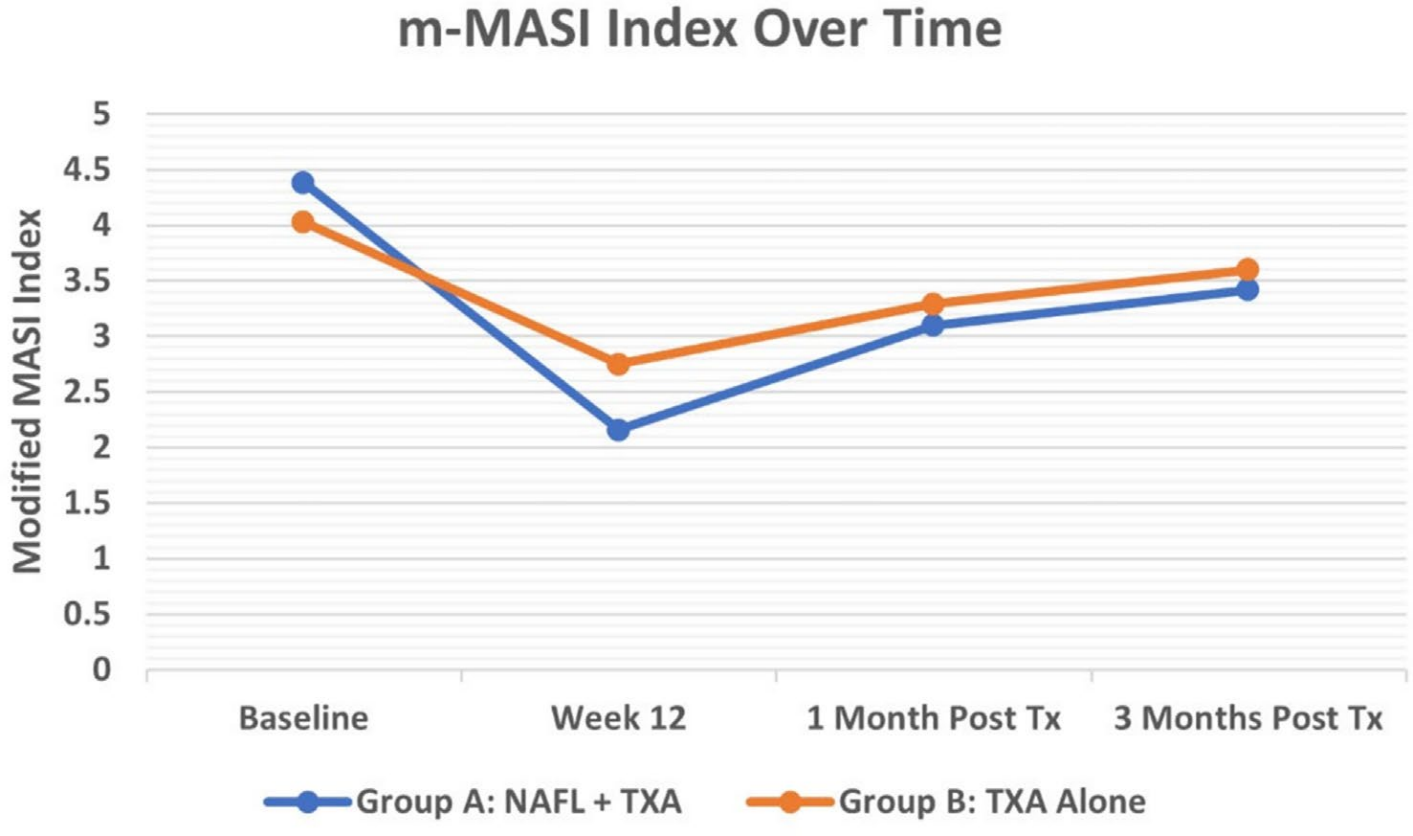
Comparison of the trend of m‐MASI index changes.

Interestingly, baseline data revealed a significant difference in the severity of melasma between the two sides of each participant's face (*p* = 0.01), with the NAFL plus TXA‐treated side (Group A) showing more severe melasma compared to the TXA alone‐treated side (Group B). Despite this higher baseline m‐MASI score in Group A, the reduction in m‐MASI scores by the end of the treatment period was significantly greater in this group, highlighting the added efficacy of NAFL in enhancing the therapeutic effects of TXA (Figure 3).

### 3.2 Physician Global Assessment

Similar to the m‐MASI score, PGA results demonstrated significant improvement in melasma following treatment in both groups, though outcomes differed in magnitude and persistence. In the NAFL plus TXA‐treated group (Group A), 40.7% of participants achieved *excellent improvement* (≥ 75%), and 37% showed *marked improvement* (50%–75%) at Week 12, reflecting a substantial therapeutic effect. However, recurrence was observed after treatment discontinuation, with PGA scores declining by the first month post‐treatment. At this time, only 7.4% of participants maintained *excellent improvement*, while 51.9% had *marked improvement*. By the third month, no cases showed *excellent improvement*, with the majority shifting to *good improvement* (37%) or *marked improvement* (25.9%), and 22.2% experienced *worsening* of their condition.

In the TXA‐only group (Group B), PGA scores were comparatively lower at Week 12, with most participants exhibiting *marked* (29.6%) or *good improvement* (29.6%). Recurrence was more pronounced in this group after treatment discontinuation, with the most common outcome being *fair improvement* (44.4%) at 1 month and 55.6% at 3 months post‐treatment. Only a small proportion maintained higher improvement levels, and worsening was also observed, though to a lesser extent compared to Group A.

**FIGURE 4:**
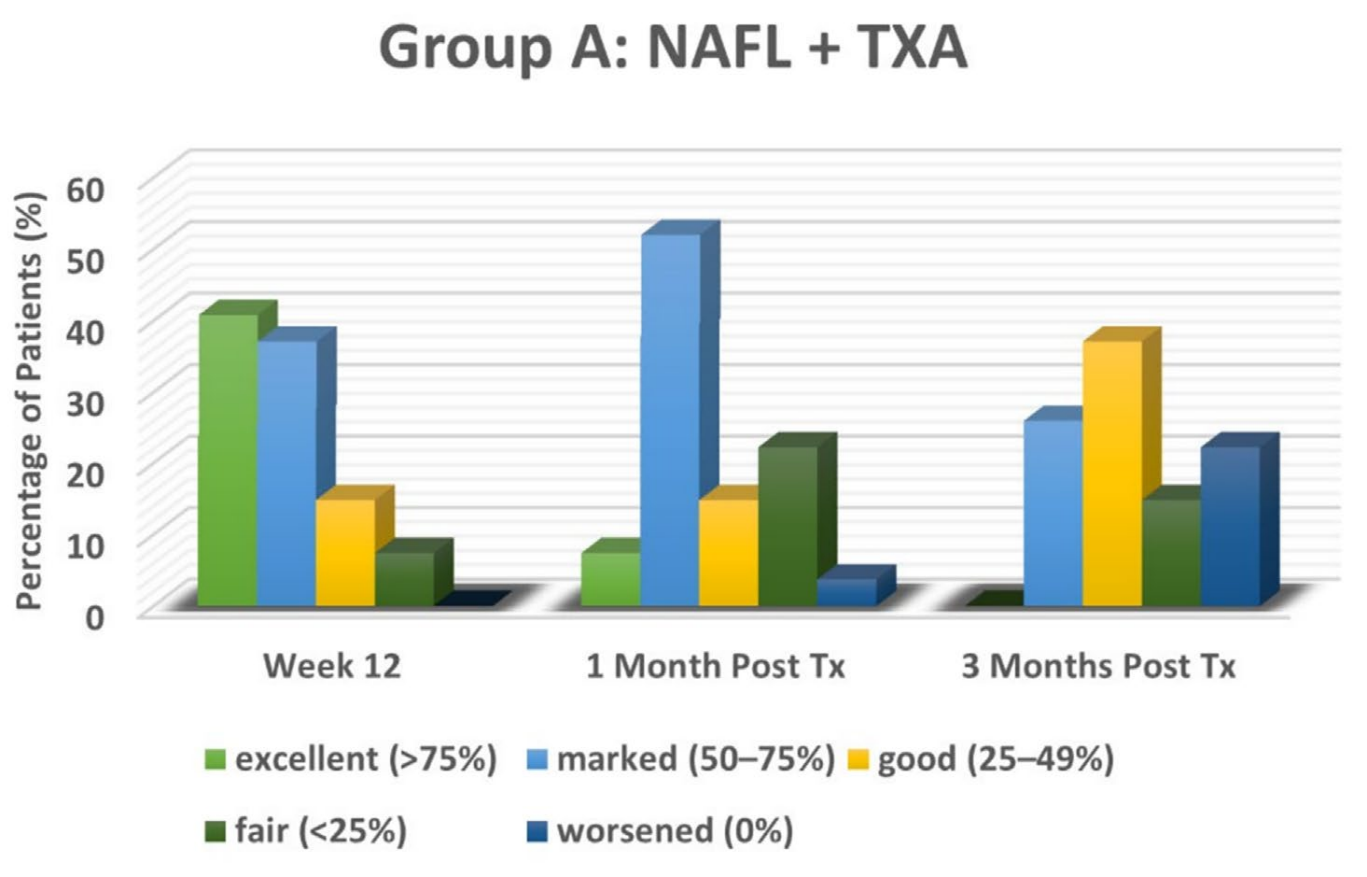
Physician Global Assessment Scores in NAFL plus TXA‐treated group (Group A).

**FIGURE 5:**
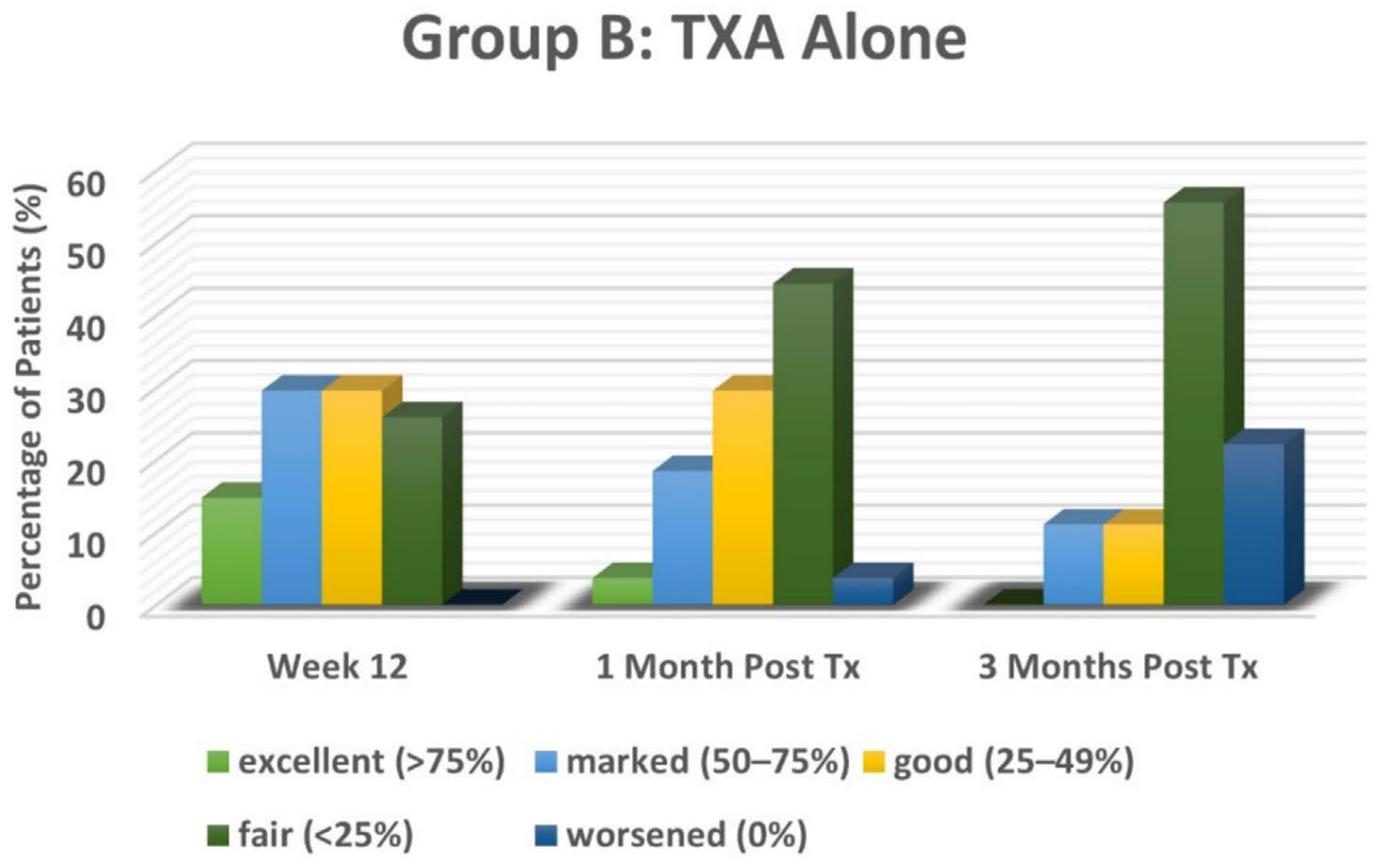
Physician Global Assessment Scores in TXA‐only group (Group B).

As illustrated in Figures 4 and 5, NAFL plus TXA treatment resulted in significantly greater PGA improvement at Week 12 compared to TXA alone (*p* < 0.001). However, by the first and third months post‐treatment, the difference between the two groups was no longer statistically significant (*p* = 0.06 and 0.07, respectively), underscoring the temporary nature of the improvement in both treatment arms. Overall, while NAFL combined with TXA provided superior short‐term results, both groups experienced recurrence after stopping treatment, emphasizing the need for long‐term management strategies.

### 3.3 Participant Satisfaction

The results demonstrated that participant satisfaction levels were notably higher in the NAFL plus TXA‐treated group (Group A) compared to the TXA alone‐treated group (Group B) at all assessment points. In Group A, at Week 12 (end of treatment), 48.1% of participants reported complete satisfaction, and 29.6% had moderate satisfaction, reflecting the significant improvement observed in participant satisfaction scores. However, satisfaction levels declined after treatment cessation. By the first month, moderate satisfaction remained high at 51.9%, though complete satisfaction dropped to 7.4%, and dissatisfaction rose to 22.2%. At the third month, dissatisfaction further increased to 33.3%, and no participants reported complete satisfaction, indicating melasma recurrence after treatment cessation.

**FIGURE 6:**
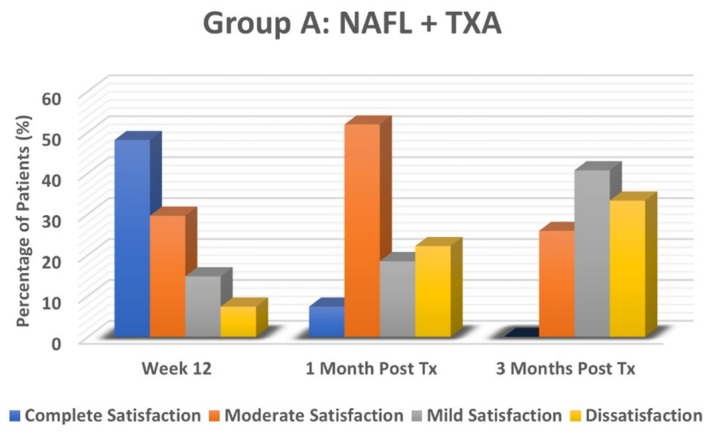
Participants Satisfaction Levels in NAFL plus TXA‐treated group (Group A).

**FIGURE 7:**
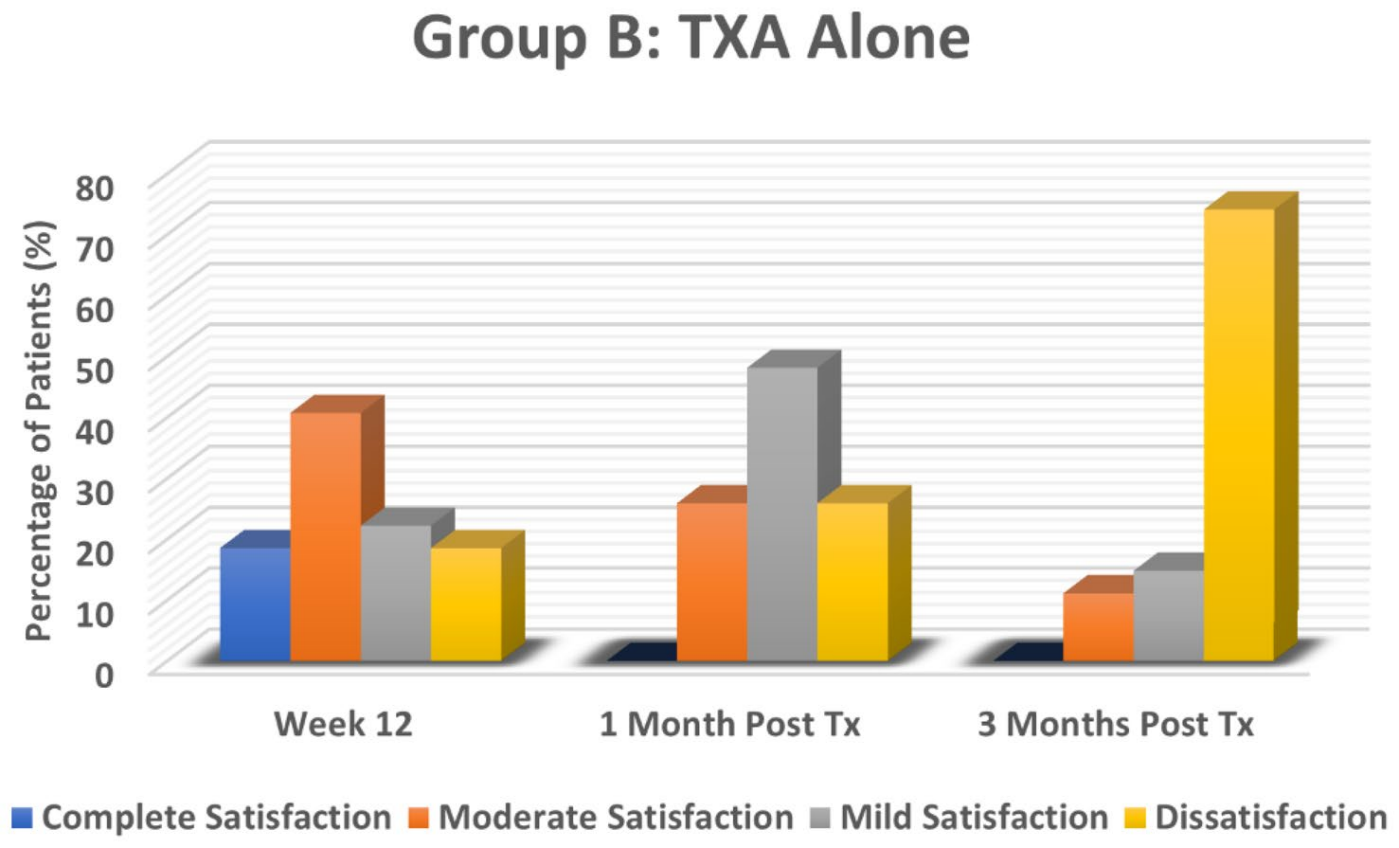
Participants Satisfaction Levels in TXA‐only group (Group B).

In Group B, satisfaction levels were initially moderate at Week 12, with 40.7% reporting moderate satisfaction and 18.5% reporting complete satisfaction. However, the decline in satisfaction was more pronounced in this group. By the first month post‐treatment, the majority (48.1%) reported only mild satisfaction, and 25.9% were dissatisfied. By the third month, dissatisfaction surged dramatically to 74.1%, with no participants reporting complete satisfaction. This highlights the superior efficacy of NAFL plus TXA treatment in maintaining participant satisfaction levels compared to TXA alone (*p* = 0.001), despite the recurrence of melasma in both groups over time. Cultural expectations surrounding laser treatments may have further influenced dissatisfaction in Group B, where some participants perceived a lack of efficacy due to the absence of laser intervention (Figures 6 and 7).

The results also indicated a significant association between m‐MASI scores and physician global evaluation results as well as participants' satisfaction levels in both groups and all assessment times (*p* < 0.05), except in the first and third months after TXA treatment, where m‐MASI scores and PGA were not associated with participants' satisfaction levels (*p* > 0.05).

### 3.4 Adverse Effects

Among the participants, only two reported mild burning and temporary redness at the site of TXA application. Notably, these individuals did not attend the follow‐up sessions and subsequently withdrew from the study. On the other hand, the laser treatment not only caused no adverse effects but also led to positive outcomes for some participants. Three participants specifically noted a reduction in fine wrinkles around the eyes and cheeks, suggesting an additional aesthetic benefit of the laser therapy beyond its intended use for melasma treatment. These findings underscore the overall safety and potential secondary advantages of the interventions used in this study.

## 4 Discussion

Melasma, a common dermatological condition characterized by hyperpigmented patches and macules, remains a therapeutic challenge due to its incomplete treatment efficacy, recurrent nature, and the high prevalence of adverse effects associated with various interventions [14]. Recently, optoelectronic technologies, such as non‐ablative fractional lasers (NAFLs), have been widely employed in the management of melasma [15, 16].

The findings of the current study revealed a marked improvement in pigmentation among participants with melasma following both TXA and NAFL plus TXA treatments. Notably, although a significant reduction in the m‐MASI scores was observed at Week 12 compared to baseline, subsequent assessments in the first and third months indicated substantial recurrence on both treated sides. Moreover, the combination treatment of NAFL plus TXA resulted in significantly greater improvement in m‐MASI scores at Week 12 compared to TXA alone, yet no significant difference was observed between the two groups during follow‐ups at the first‐ and third‐months post‐treatment.

These findings underscore the transient efficacy of both therapeutic modalities. While significant clinical improvement occurred during the active treatment phase, the recurrence of pigmentation post‐treatment highlights the persistent challenge of achieving long‐term remission in melasma management. Importantly, the study demonstrated that NAFL, despite its short‐term benefit, does not effectively prevent post‐treatment melasma relapse.

Topical TXA is recognized as a safe and well‐tolerated treatment for melasma, with fewer side effects than other depigmenting agents such as hydroquinone (HQ). For instance, the trial conducted by Atefi et al. [17] comparing topical TXA 5% with HQ 2% over a 12‐week period in women demonstrated higher satisfaction rates and fewer adverse effects in the TXA group. However, monotherapy with topical 5% TXA alone often fails to adequately reduce pigmentation, particularly in individuals with darker skin types [13]. This limited efficacy may be attributed to insufficient delivery of the active compound to the target dermal layers. In contrast, deeper and more uniform delivery through techniques such as microneedling has shown promise in enhancing the therapeutic outcomes of TXA in melasma [18].

In light of this, we hypothesized that combining NAFL with TXA may enhance transdermal delivery and improve treatment outcomes. The present study supports this hypothesis, demonstrating that the combination therapy is beneficial in the short term; however, its long‐term efficacy remains limited. In agreement with our findings, Tourlaki et al. [19] reported that NAFL, when combined with a triple‐combination cream (TCC), showed significant short‐term efficacy in TCC‐resistant melasma cases, though sustained benefits were restricted to a small subset of participants.

These observations suggest that although the combination of NAFL and TXA is superior to TXA alone in the short term, complete or near‐complete remission of melasma in the long term is achieved in only a limited number of cases. Mechanistically, NAFL facilitates transepidermal melanin removal by generating microscopic thermal zones, while TXA inhibits plasmin activity in keratinocytes, thereby suppressing prostaglandin production and UV‐induced melanogenesis. This dual mechanism, particularly through epidermal plasmin suppression, helps prevent recurrent hyperpigmentation [20]. Furthermore, NAFL may enhance TXA penetration by creating microscopic channels, potentially increasing its absorption [21, 22].

It is noteworthy that the trends observed in mean PGA and participant satisfaction closely mirrored the m‐MASI score outcomes. While both treatment groups experienced recurrence in the long term, the NAFL plus TXA group demonstrated significantly higher satisfaction levels. This could be attributed to the perceived superiority of laser‐based interventions, possibly due to their additional cosmetic benefits such as wrinkle reduction through enhanced collagen production and reduced collagen degradation [23], or the impression of greater efficacy due to their advanced technological nature.

The observed discrepancy between quantitative (m‐MASI) and qualitative (satisfaction) outcomes may be due to methodological differences. While the m‐MASI offers a precise, objective assessment of melasma severity, satisfaction ratings rely on subjective, categorical choices (e.g., dissatisfied, mildly satisfied, moderately satisfied, or completely satisfied), which lack granular precision. Additionally, cultural expectations or preconceived beliefs about treatment efficacy could have influenced participant‐reported satisfaction, contributing to the observed differences between groups A and B.

## 5 Conclusion

In conclusion, both topical 5% TXA and NAFL 1540 nm demonstrated substantial short‐term improvements in melasma severity. However, the combination therapy yielded superior results during the active treatment phase. Despite this, neither treatment was effective in significantly preventing melasma recurrence in the long term. Further efforts are required to develop more durable therapeutic options.

## 6 Limitations and Future Directions

This study has several limitations. The small sample size restricts the generalizability of the findings and limits the ability to conduct subgroup analyses based on skin type or melasma subtype. The 3‐month follow‐up period may be insufficient to evaluate long‐term recurrence, considering the chronic nature of melasma. Subjective outcome measures such as satisfaction scores are prone to recall and expectation bias, especially in interventions involving laser therapy. The lack of a placebo or sham‐controlled group limits the ability to isolate treatment effects, and the absence of participant blinding introduces potential bias. Moreover, no histological or imaging assessments were performed to confirm clinical observations, and variations in adherence to topical treatment and sun protection may have influenced the results.

Future research should address these issues by conducting larger, multicenter randomized trials with longer follow‐up durations of at least 6–12 months. Incorporating placebo arms, blinding protocols, and objective adherence monitoring tools would enhance study rigor. Use of standardized, quantitative assessment methods alongside subjective evaluations is recommended to reduce bias. In addition, mechanistic studies employing biopsies, non‐invasive imaging, or molecular markers could clarify the pathways through which NAFL and TXA act. Investigating advanced drug delivery systems such as microneedling or nanoformulations may also help improve treatment durability and efficacy in melasma.

## Author Contributions

A.H.E., P.N., and A.R. designed the research study. Z.S., M.K.M., M.H., and A.E. performed data collection. A.R., M.K.M., and Z.S. analyzed and interpreted the data. M.H., A.E., and A.R. prepared the manuscript draft. A.R., A.H.E., and P.N. reviewed, edited, and approved the final manuscript. All authors have reviewed the manuscript and provided their approval for its submission.

## Conflicts of Interest

The authors declare no conflicts of interest.

## Data Availability

The data that support the findings of this study are available from the corresponding author, Ala Ehsani, upon reasonable request.
